# COVID‐19 shines a light on health inequities in communities of color: A youth‐driven photovoice inquiry

**DOI:** 10.1002/jcop.22866

**Published:** 2022-04-20

**Authors:** Astraea Augsberger, Noor Toraif, Adrienne Young, Noelle C. Dimitri, Rosaylin Bautista, Ja'Karri Pierre, Catherine Le, Osasenaga Idahor, Calvin Jusme, Katherine A. Gergen Barnett

**Affiliations:** ^1^ School of Social Work Boston University Boston Massachusetts USA; ^2^ Department of Family Medicine Boston Medical Center Boston Massachusetts USA

**Keywords:** antiracist practice, community based participatory research, COVID‐19, health equity, photovoice, Youth Advisory Board

## Abstract

This manuscript reports on a youth‐driven health assessment engaging youth of color in identifying community health priorities during the coronavirus disease 2019 (COVID‐19) pandemic. Photovoice, a participatory visual ethnographic health assessment strategy, was used to explore the question: *What does health or healthiness mean to you and/or your community?* Youth captured images that represented their priorities. The photos were discussed using the SHOWed framework and analyzed thematically. Four themes related to community health were identified. Additionally, youth captured their narrative of COVID‐19 as “a revealing force that highlights systemic inequities, driving individuals and communities to both cultivate their resilience and take healthcare into their own hands in response to government and policy level failures.” Youth are acutely aware of the historical and structural inequities that create multi‐level barriers to healthcare access. Health inequities existed long before the pandemic, but the current crisis requires us to examine ways to transform the healthcare landscape moving forward.

## 1 INTRODUCTION

Youth movements for social justice are growing across the United States and worldwide, tackling important issues with everything from climate change, to racial justice, to education access, and to gun control (Witt, 2019). Young people are bringing fresh energy, ideas, and direction to problems they are inheriting from generations before them. Youth participation in decision‐making has multiple benefits to communities, organizations, and society. It contributes to a healthy democracy, as youth are an important constituency impacted by policy and programs (Collins et al., 2016). Youth bring up‐to‐date and relevant information that can lead to policy and programs aligned with community needs and priorities (Sprague Martinez et al., 2018). In the realm of healthcare, youth participation can lead to greater patient understanding, patient engagement in services, and enhanced trust in services (Brown et al., 2016). It can also promote patient and community empowerment (Sprague Martinez et al., 2020).

Recognizing the benefits of youth engagement and leadership, the present study captures the perspectives and experiences of youth on the issue of individual and community health. The manuscript describes the work of Youth Advisory Board (YAB) members, supported by a team of adults from Boston University School of Social Work and the Boston Medical Center Family Medicine Department, to uplift the experiences of youth of color^1^ who have been historically marginalized by the healthcare system. It describes Photovoice, a participatory visual ethnographic health assessment strategy, that took place during the height of the COVID‐19 pandemic to explore the question: *What does health or healthiness mean to you and/or your community?* It presents visual data collected and analyzed by youth researchers in partnership with adult facilitators. The study is timely in that research demonstrates that COVID‐19 disproportionately impacts low‐income and communities of color (Ash et al., 2021). Further, distrust in medical professionals and medical systems results in, among other things, worse outcomes for patients—further perpetuating health inequities (Alpers, 2018). Youths' understanding of individual and community health, especially during the COVID‐19 pandemic, is thus critical to tackling health equity.

### 1.1 Photovoice methods

Developed by Wang and Burris (1994) and originally known as “photo novella,” photovoice is a method designed to allow people to “document and discuss their life conditions as they see them” through photography (p. 171). While anyone can theoretically be a subject of this method, its original design was inspired by Paulo Freire's concept of *education for critical consciousness* (Freire, 1970). Critical consciousness aims to empower marginalized people who lack power, wealth, and status to critically reflect on their own experiences and conditions, as well to challenge and transform those conditions and the social systems that uphold them (Budig et al., 2018; Liebenberg, 2018; Wang & Burris, 1994). Photovoice is thus a form of participatory research guided by an ethical imperative to democratize knowledge production among the marginalized and oppressed, and to amplify their voices so as to facilitate socially transformative action (Budig et al., 2018; Liebenberg, 2018). Participatory research aims to engage community members in each stage of the research process, from study design, to data collection, to data analysis, to the dissemination of findings. “Participatory approaches can be a particularly strong way to ensure marginalized communities, including people of color and people with low economic status as well as youth, are actively engaged in research” (Teixeira et al., 2021, p. 3).

During photovoice, participants are given cameras, taught how to use them as needed, and asked to photograph images of their choice about their lived experiences in response to broad prompts or questions that researchers and participants have come to a consensus on through discussion (Liebenberg, 2018; Packard, 2008; Wang & Burris, 1994). The method rests on the assumption that the images the participants choose to capture are not simply random, but rather are shaped by cultural signifiers, as well as social, communal, and personal values, expectations, and meaning‐making frameworks (Harper, 2002; Liebenberg, 2018). After the images are produced by individual participants, the method requires participants and researchers to collectively interpret images as a group to co‐construct the meaning of these images (Liebenberg, 2018). The role of researchers is to guide this co‐construction of meaning by asking broad questions to “identify the problem or the asset, critically discuss the roots of the situation, and develop strategies for improving the situation” (Wang, 1999, p. 190).

### 1.2 Photovoice as a mechanism for participant empowerment

Photovoice researchers have applied this method to successfully achieve these goals of transforming participant understandings and facilitating action towards social change in a variety of ways. Budig et al. (2018) found that female participants in a prior photovoice project reported improved knowledge of their community, greater critical awareness of their community, a more empowered self‐perception, and expanded network connections to their community, the media, decision‐makers, and researchers. In the original Wang and Burris (1994) study, the authors found similar effects for both changes in consciousness and in actions for potentially changing policy among rural women in Yunnan province in China. Strack et al. (2004) adapted photovoice to a youth after‐school program and found that it helped the youth develop their personal and social identities, as well as their own social morality. In a review of the literature on photovoice, Catalani and Minkler (2010) found that photovoice projects were highly variable in their goals, but 60 percent of them reported producing an action component, and the more participatory projects tended to produce a greater understanding of the community's assets and needs, as well as a greater sense of empowerment.

**Figure 1:**
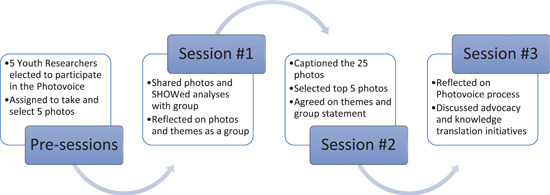
Photovoice sessions

### 1.3 Photovoice as an antiracist modality

Photovoice has also been used as an antiracist modality. Goessling (2018) used photovoice to evaluate the experiences of low‐income urban high schoolers who engaged in a photovoice project and the development of their critical consciousness. While the youth had been subject to a deficit‐focused and racist narrative of the inadequacies and troubled nature of their school and community, principally by the Gates Small Schools Initiative, it was through their photovoice project that the youth developed counter‐narratives that affirmed the beauty and assets of their community, themselves, and their school. Markus (2012) used photovoice in the context of an American Indian community to address problems of HIV, sex education, and social determinants of health. Through photovoice, youth and community members were able to develop antiracist storytelling for social justice and build their community's capacity to challenge their conditions and address their identified problems. Stanton and Hancock (2021) used photovoice to evaluate and cultivate the critical consciousness and antiracist/anticolonial pedagogy of preservice elementary social studies teachers. They found that these teachers needed greater sustained engagement during preservice teacher training with opportunities to develop critical antiracist consciousness, self‐definition as teachers, and self‐transformation as teachers of social justice and that photovoice projects can provide such opportunities.

### 1.4 Theoretical framework: The socioecological model

The socioecological model was used in the photovoice project as a sensitizing framework for YAB members to collect and analyze data related to health in their life and communities. The socioecological model was developed by Bronfenbrenner (1977) as an ecological systems theory to explain how the conditions, systems, and relationships in which an individual lives affects their development and wellbeing throughout the course of their life. In its application to the study of health, the socioecological models examine how individual, relationship, community, and society level factors overlap and interact with each other to determine individual and population health outcomes (Centers for Disease Control and Prevention, 2016). Factors in the socioecological model may be further delineated from these four factors (individual, interpersonal, community, and societal) to five or six factors depending on the complexity of the analysis and scale of the inquiry under investigation (Bronfenbrenner, 1977; Scarneo et al., 2019). For example, McLeroy et al. (1988) developed a highly influential five‐level socioecological model that evaluates how health behaviors are shaped by individual, interpersonal, institutional, community, and public policy factors.

Socioecological models have been particularly useful for research examining health inequities. As Reifsnider et al. (2005) note, socioecological models are useful for identifying and understanding health disparities between groups in a population, because they allow researchers to analyze how different combinations and scales of factors may intersect to produce particular kinds of inequities for different populations. Stunted child growth, for instance, maybe a result of interpersonal relationship factors for one child, but for another it may be a result of a lack of nutrition in their home neighborhood because of social or policy level factors (Reifsnider et al., 2005). To analyze disparities in Black and white infant mortality, socioecological models have been used to account for layered relevant factors at different levels, including poverty, internalized racism, institutional racism, stress from perceived racial discrimination, and lack of access to quality healthcare (Alio et al., 2010). Socioecological models provide levels of complexity and specificity that are useful for evaluating the various interconnected elements that produce health inequities.

### 1.5 Study context

This photovoice was part of a larger multimethods project run by the Boston University School of Social Work and the Boston Medical Center Family Medicine Department aimed to (1) develop a YAB consisting of youth of color, aged 16–25, from Boston, USA; and (2) to engage YAB members in a data‐driven health promotion campaign. The study protocol was approved by the Boston University Institutional Review Board and funded by the Boston University Clinical & Translational Science Institute.

YAB members engaged in various sessions throughout the year‐long project (August 2020–June 2021), including a 3‐day orientation, weekly team meetings, and weekly training sessions. During the orientation and training sessions, YAB members completed activities such as: (1) conducting a socioecological brainstorming activity to determine the multilevel factors that influence individual and community health; (2) participating in training on community‐based participatory research methodology, including photovoice, and youth–adult partnerships; and (3) attending lectures on health equity and healthcare service delivery.

The YAB members were eight youth of color between the ages of 16 and 25 who participated in all orientation, training, and weekly meetings. YAB members were selected from diverse neighborhoods across Boston, Massachusetts, in the United States; three self‐identified as male (37%) and five self‐identified as female (63%). Youth self‐identified racially as Latinx/Hispanic (*n* = 2), Vietnamese/Chinese (*n* = 1), Black/Asian (*n* = 1), Black/African American (*n* = 2), Black/Nigerian‐American (*n* = 1), and Afro‐Caribbean (*n* = 1). Throughout the course of the project, the YAB members engaged in multiple research activities that allowed individuals to pursue the research methods that most interested them. All eight YAB members participated in weekly team meetings building consensus around their definitions of individual and community health.

## 2 MATERIALS AND METHODS

The study used photovoice methods (Wang, 1999) to explore youths' individual and community perceptions of health. Due to the COVID‐19 pandemic, the method was adapted to an online format that included the use of the Zoom platform for all sessions. Data were collected in the form of photographic images, captions, and voice recorded group sessions. Data were critically analyzed thematically in the context of the youths' lived experiences. Adopting a participatory research approach provided an opportunity to engage a small number of youths, explore how they conceptualize health, and empower them to share their individual and community perspectives (Teixeira et al., 2021; Wallerstein & Duran, 2006).

### 2.1 Authors positionality statement

Before presenting the methods and findings, and in the spirit of reflexivity, we would like to acknowledge who we are as authors and how we approach this study. The adult researchers identify as female (*n* = 5), Middle Eastern/North African (*n* = 1), White/Native American (*n* = 1) and White (*n* = 3). We share a commitment to addressing racial health equity. We bring combined expertise in participatory methods and antiracist practice, youth engagement, health promotion, and group facilitation. Several authors have extensive experience collaborating with youth in research and practice. The youth researchers bring lived experience as youth navigating individual and community health during the COVID‐19 pandemic and bring additional expertise as YAB members.

### 2.2 Research team

All eight YAB members were given the opportunity to participate in the photovoice. Five of the eight YAB members self‐elected to participate, while the remaining three youth pursued other projects related to their end goal of health promotion for the Boston Medical Center Family Medicine Department. The youth research team (herein referred to as “youth researchers”) self‐identified as male (*n* = 3), female (*n* = 2), Latinx/Hispanic (*n* = 1), Vietnamese/Chinese (*n* = 1), Black/African American (*n* = 1), Black/Nigerian‐American (*n* = 1), and Afro‐Caribbean (*n* = 1). The five youth researchers reported their progress and findings back to the full YAB during their weekly check‐in meetings.

The adult research team consisted of three facilitators and two principal investigators of the study. Although adult facilitators ran the photovoice sessions, the data collection, analysis, and findings were driven by the youth research team. For example, youth researchers controlled the subject of photos they captured and selected which ones to share with the larger group. The youth researchers uploaded their own photos to a shared Google drive, created a Google slide presentation, and each shared their slides in Zoom. The adult researchers were intentional in posing open‐ended questions for youth reflection and discussion, allowing time for youth to wrestle with their ambivalence and come to their own conclusions. The adult researchers played a supportive role, as opposed to directing the youth discussions. The facilitator with expertise in photovoice also provided feedback as needed to the adult researchers using the Zoom chat feature to ensure the voices of the youth researchers were prioritized.

### 2.3 Procedures

In December 2021, an adult researcher with expertise in photovoice methods facilitated a 60‐minute photovoice training with the youth researchers and other members of the adult research team using Boston University's secure Zoom platform. Youth researchers received in‐depth training on the photovoice method, and the facilitator shared an example of a previous photovoice research study she conducted (Dimitri, 2021). The facilitator briefly reviewed research ethics and confidentiality including the importance of gaining written consent when taking photos of people. During this pre‐session, youth researchers were asked to take a series of photos using their cell phones over a period of approximately 3 weeks. Due to the time restrictions of the sessions, youth researchers were instructed to choose up to five photos to share with the larger group. The youth researchers were instructed to upload their five photos to a secure Google drive site to present to the group during the first session. Participants were given agency and creative choice in deciding what types of photos to capture; this particular group chose to not photograph individuals and instead captured *What does health or healthiness mean to you and/or your community?* in other parts of the social environmental context. As a result, participants minimized potential ethical concerns using their cell phones.

In January 2021, five youth researchers participated in three 90‐minute sessions using Boston University's secure Zoom platform. The sessions were largely facilitated by one adult researcher with expertise in photovoice methods. The other two adult facilitators assisted with the smaller group discussions, taking notes, and supporting the youth researchers. Sessions were audio‐recorded with the youth researchers' permission and transcribed verbatim by Zoom.

### 2.4 Photovoice sessions

Figure 1 in the first session youth researchers and adult researchers engaged in an icebreaker activity and discussed group agreements to build rapport and trust in the group space. The ice breaker activity allowed youth researchers and facilitators to share their names, pronouns, one thing that they could see in their virtual space that made them happy, and one thing they were looking forward to in the meeting. The group agreements mirrored the group agreements of the YAB overall including sentiments such as, “assume best intentions,” “take space and make space,” “confront the idea not the person,” and “listen to understand.”

Then youth researchers shared their photos with the group using the screen‐share function in Zoom, and individually described their photos using the SHOWed method, a series of questions to encourage deeper reflection on how social, cultural, and/or contextual factors impact how youth of color conceptualize what health means to them (Wang & Burris, 1997). The questions included:

(1) What do you **S**ee here?
(2) What's really **H**appening here?
(3) How does this relate to **O**ur lives?
(4) **W**hy does this problem, concern, or strength **E**xist? and
(5) What can we **D**o about it?

A total of 25 photos were presented by the five youth researchers. Drawing from the SHOWed questions (Shaffer, 1991), each youth researcher narrated the meaning and story behind each of their photos to the group, which included their peers and the adult research team. Next, the youth researchers were asked to share their reactions to seeing the photos as a group, including what they found interesting and what stood out to them. The adult facilitators also shared their reactions to the photos once the youth researchers had the opportunity to comment. Then, the youth researchers were invited to turn their cameras off and write a brief memo reflecting on their personal reactions, thoughts and feelings, evoked by the images and accompanying stories (Charmaz, 2014). Although we did not employ grounded theory methods, we relied on memoing to provide the youth researchers with an opportunity to reflect in greater depth on the photos and group discussions. In the final activity of the first session, the youth researchers were divided into two groups randomly and asked to consider the primary themes emerging across the photos and group discussion. One adult facilitator joined each group and took detailed notes and participated minimally in the discussions. The larger group re‐convened and youth researchers from each group were asked to share what came up in their small group and to comment on the larger story emerging from their discussions. Youth researchers described common themes reflected across the groups. Youth researchers also recalled previous YAB discussions about individual and community health, and noted some absent themes (e.g., American culture, power, and influence, disbelief in COVID‐19).

One week later in the second session, the facilitator reviewed the session agenda and youth researchers participated in an ice breaker activity. The ice breaker activity allowed youth researchers and facilitators to share how they were doing, and if they would prefer to give up their sense of smell or taste and why. Next, youth researchers were divided into two groups randomly with one adult facilitator in each group to take notes. The first group (*n* = 2) captioned photos 1–10 in the slideshow while the second group (*n* = 3) captioned photos 11–25. Each group captioned the assigned photos that were not necessarily their own. Next, the larger group reviewed the entire slideshow of images with their captions. Youth researchers took turns reading the captions aloud to the group. Youth researchers were encouraged to voice feedback about the captions, especially if they did not agree with the captions on their own photos, and were invited to share any changes they felt were needed. Next, youth researchers were asked to further review the larger group of 25 photos and to identify five key photos reflecting the larger story the group identified in the data. Each youth researcher took time to independently review the entire slideshow and added a unique symbol using the annotation tool in Zoom to identify their selection of five photos. Youth researchers each selected a different symbol and anonymously selected five photos. Youth researchers were reminded they could choose to vote for one photo or multiple photos. One of the facilitators tallied the results and presented the five photos with the most votes in a new slideshow. Youth researchers were asked to reflect on this process of reviewing and then refining their selection of certain photos. Youth researchers also considered any emerging themes among the selected images.

Consistent with thematic analysis (Braun & Clarke, 2006), youth researchers were asked to brainstorm themes from the individual photos and across the photos, considering how the photos connected with the research question: *What does health or healthiness mean to you and/or your community?* As a group, youth researchers shared their thoughts with each other and gradually refined their understanding of the central themes and story until they reached a unanimous decision on the content and language. Facilitators checked in for agreement among youth researchers who were asked to comment both verbally and nonverbally. Through in‐depth discussion and drawing from the identified themes, youth researchers reached a consensus on the larger narrative represented in the photos. In closing the second session, youth researchers were asked to again engage in a memoing activity writing brief notes and reflections capturing their reactions to the photos and their experience in the photovoice overall.

Two weeks later, in the final session, youth researchers were encouraged to reference their memos and share their thoughts on the photovoice process, and how the agreed‐upon themes had been sitting with them. Youth researchers shared reflections on their similarities and differences, and how they had been thinking about each other's photos over the previous weeks. Next, youth researchers were invited to brainstorm future opportunities to share their photovoice analysis and images in the community through action and advocacy initiatives. Youth researchers identified wanting to write a newsletter for the hospital to share through community engagement initiatives and to participate in the writing of an academic manuscript.

## 3 RESULTS

Four themes related to youth and community health were identified through the digital images and discussions, including: (1) taking health into our own hands; (2) toxic productivity culture; (3) high cost of personal health resources, and (4) inequitable health policies and services. The context of COVID‐19, with everything being shut down, people working and going to school from home, and a lack of responsive systems and policies, were important themes that arose in the youth discussions. Below the digital images and themes are discussed further.

### 3.1 Taking health into our own hands

**Image 1:**
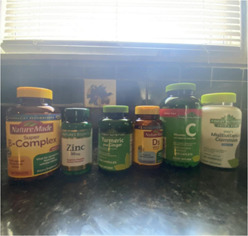
We have medicine at home.

Youth researchers discussed the historical and current lack of trust with the healthcare system in communities of color in the United States and their practice of using familial cultural remedies to prevent illness and/or manage pain. Image 1 “*We have medicine at home*” is a picture of vitamins that one youth researcher's mother has the family take on a daily basis to build their immune system and prevent the family from getting sick with COVID‐19. The youth researchers discussed specific examples such as the Tuskegee Syphilis study and the long history in the Black community in the United States of being abused by the healthcare system contributing to a lack of trust, and the resultant hesitancy to take the COVID‐19 vaccine. Youth researchers further reflected on the ways in which individuals and communities of color “took healthcare into their own hands” by using their family and cultural health management strategies. Youth researchers discussed the salience of maintaining their cultural healthcare practices among generations and the importance of viewing them as holistic and integrative medicine.

**Image 2:**
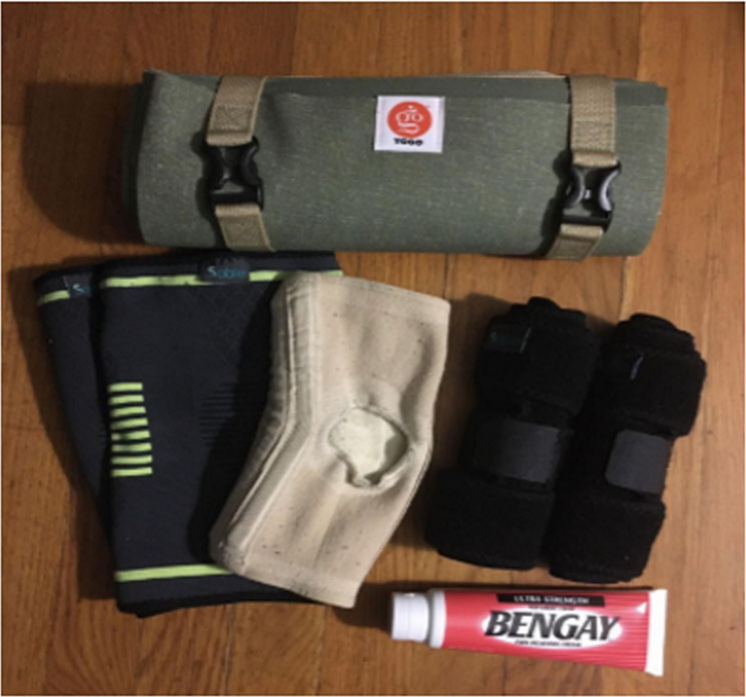
Figuring out my own treatment when my pain is dismissed.

Youth researchers reported feeling dismissed by healthcare professionals. Image 2 “*Figuring out my own treatment when my pain is dismissed*” captures a yoga mat, knee braces, knee sleeves, and Bengay. The youth researcher described having bad knees since middle school. When she discussed the concern with her primary care doctor, they told her it was a common issue for teenagers to have bad knees. The youth researcher felt the doctor disregarded her pain and she had *no other option* but to employ her own pain management strategies. The youth researcher reported that “many people have experiences with their concerns being dismissed (by healthcare professionals) and it's discouraging.” She indicated that potential reasons for the dismissal could include age, gender, race, and time constraints on the part of healthcare professionals. Youth researchers discussed the importance of healthcare professionals taking time to listen to youth of color and develop a relationship that fosters honest and direct communication.

### 3.2 Toxic productivity culture

Youth researchers discussed the use of self‐care practices in a society that doesn't prioritize health, mental health, and well‐being. During the height of the COVID‐19 pandemic, when schools and workplaces shut down, there remained the expectation of high performance, especially as it related to school and work. There was a lack of separation between work and home and trauma caused by the multiple losses related to the pandemic, which further contributed to a “toxic productivity culture” that demanded work continue. As an example, one youth described her “home workspace” which was located in her bedroom with a laptop, books, and other study materials. There was no separation between where she slept and where she worked which was toxic to her overall well‐being.

**Image 3:**
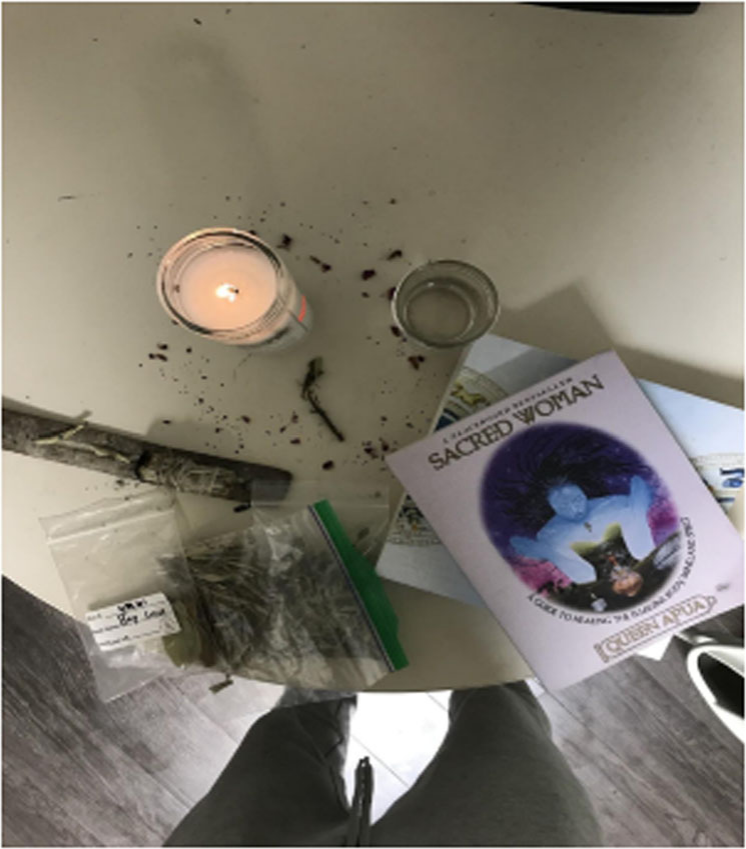
Spirituality is self‐preservation.

To cope with this “toxic productivity culture,” youth researchers discussed the self‐care practices they used to promote their own health and mental health, including spirituality, exercise, and listening to music, among others. Youth researchers shared various photos reflecting these practices. Image 3  **“** *Spirituality is self‐preservation*” is a traditional altar, with a book on spirituality, a candle, and herbs for a ceremony. The youth researcher discussed how the practice of spirituality is a mechanism he uses to connect with his African ancestors and blessed spirits. This practice prevents him from feeling isolated and alone during a time when he is not able to be physical with his family or friends.

### 3.3 The high cost of personal health resources

**Image 4:**
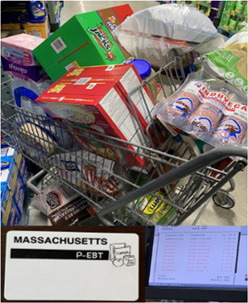
A shopping cart full of food and other necessities paid for using the Massachusetts P‐EBT card.

Youth researchers discussed how the COVID‐19 pandemic and the concurrent Black Lives Matter Movement has opened people's eyes to the loss and racial inequity in this country. The pandemic “shed a light” on unequal access to resources such as time, energy, and finances. Image 4  “*A shopping cart full of food and other necessities paid for using the Massachusetts P‐EBT card*” shows a shopping cart full of food and other necessities paid for using Supplemental Nutrition Assistance Program (SNAP) benefits through the Massachusetts Pandemic (P‐EBT) card, that provides financial assistance to help families with children who were receiving free or reduced‐price school meals. The benefit was provided during the school year and over the summer months. The youth researcher who took the photo discussed how pre‐pandemic many families relied on free or reduced meals at school because parents are working all day, cannot afford to buy food, and/or some children don't have parents they can rely on to give them food. While schools provided food during the pandemic, there were issues related to access. For example, the food was only at certain sites and/or on certain days, which meant not all kids received three meals a day. The youth researcher noted that “there should be a way that kids and their families can easily access three meals a day, every day.”

### 3.4 Inequitable health policies and services

Youth researchers discussed the unequal distribution of green space and disparate impacts of COVID‐19 on communities of color. One youth stated, “We all lost access or a willingness to want to go outside because of COVID concerns. Policy shapes and dictates green spaces and how accessible they are to people and we shouldn't take that for granted.”

**Image 5:**
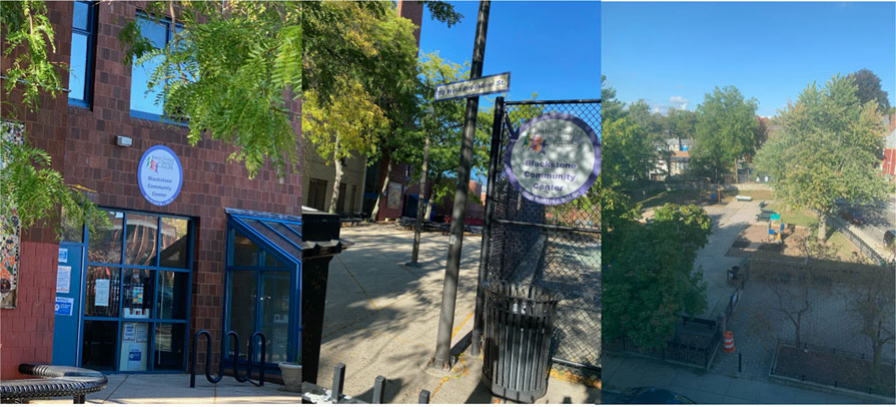
Community unity, but there's no community.

Image 5  “*Community unity, but there is no community*” is a playground and local community center. The youth researcher reflected on how they were once filled with children playing, allowing them to relax, exercise, connect with others, and relieve stress, but during the height of the COVID‐19 pandemic the playground remained empty and unused. COVID‐19 impacted daily life and people were not able to do what made them healthy and happy. Youth researchers discussed how COVID cases were rising in the city and the lack of emergency preparedness and guidelines in place. They reiterated the need for more guidance/directives from policymakers, including holding people who don't follow public health guidelines accountable.

### 3.5 COVID‐19 group statement

As seen through the photovoice digital images and ensuing youth researcher discussions, youth researchers identified multi‐level themes—on the individual, family, and community levels. Upon deeper discussion, the youth researchers developed a group narrative focused on system and policy level failures. The youth collectively developed this statement to summarize their work:

*COVID‐19 is a revealing force that highlights systemic inequities, driving individuals and communities to both cultivate their resilience and take healthcare into their own hands in response to government or policy level failures*.

While developing their collective narrative, one youth researcher stated:

*I see healthcare as multi‐level. People pay attention to the personal and small community level but taking a step back to look at the greater community (everyone) is not considered as much. You need to have the time and place to take a step back to reflect. It is important that individuals don't take on the burden of the healthcare system failing their communities*.

## 4 DISCUSSION

The youth researchers identified four themes related to what health and healthiness means to them and their community: taking health into our own hands, toxic productivity culture, high cost of personal health resources, and inequitable health policies and services. While the digital images and reflections using the SHOWed method were initially focused largely on the individual, family, and community levels, the group discussions and statements identified system and policy level failures that disproportionately impact the health of communities of color and lead them to “take healthcare in their own hands.”

Health inequities in communities of color have long existed before the pandemic (Ottersen et al., 2014). However, COVID‐19 merely highlighted and exacerbated these deep inequities (Ash et al., 2021). Not only were these health inequities driven by the lack of critical resources and protections in communities of color, but also, and perhaps just as importantly by the way that the pandemic has been messaged—further driving medical distrust in communities of color. This plays out in the data. In October of 2020, 7 of 10 Black Americans stated they're treated unfairly by the healthcare system and 55 percent say they distrust it (Fletcher, 2020). Further, people who say they mistrust healthcare organizations are less likely to take medical advice, keep follow‐up appointments, or fill prescriptions; thus often making them feel like they need to take their health into their own hands (LaVeist et al., 2009).

Youth researchers experienced this on a personal level—having to manage the stress that the pandemic brought to themselves and their families—as well as on a structural level. They acknowledged the years of inequitable policy that have disrupted access to health for communities of color (Williams & Mohammed, 2013). Young people of color recognize the historical and current structural and racial inequities that create a barrier to health access in Boston and across the United States (Williams et al., 2019). They discussed how, from their perspective, even well‐intentioned policies and practices addressing health equity do not go beyond the surface level to influence positive health impacts for youth and communities of color.

### 4.1 Implications

The results of this photovoice inquiry have implications for the future of healthcare policy and research. The themes highlighted by the youth researchers demonstrate that youth are not only thinking about health equity but also grapple with these issues on multiple socio‐ecological levels. In particular, youth researchers highlighted the structural racism underpinning health policy and programs and thereby negatively impacting individual and community health. Youth researchers also discussed the need to take their health into their own hands, given the healthcare system's failure to reach them, the historical lack of trust with the medical communities, and the lack of racially and culturally responsive services. One youth researcher discussed healthcare providers disregarding her pain, another described their parents and other family members using herbal remedies and several vitamin combinations to prevent illness. In thinking about some of the most pressing healthcare needs moving forward, the youth researchers highlighted the need for access to inexpensive and healthy foods, access to green space, integrative healthcare, and prioritizing self‐care.

While our data show that youth have a complex understanding of the multiple determinants of health, they perceive their voices are often not heard or valued. How can we shift this tide? From a health system practice perspective, including youth voice and leadership in shaping clinical initiatives and community engagement is critical (Sprague Martinez et al., 2020). It is clear from this photovoice project that youth understandably hold some of the present‐day and historical medical distrust. Truly listening and engaging youths' perspectives is thus critical in the medical system's efforts to build medical trust and bridge the gap between community fear and the communication of important health information (e.g., vaccine hesitancy; Fisher et al., 2020).

Furthermore, health leaders and government representatives must do more than simply recognize inequity; they must intentionally shift power to marginalized members of the healthcare system to influence sustainable and equitable access to healthcare (Sprague Martinez et al., 2018). They would therefore do well by consulting youth as they are thinking about the implications of either long‐held or new health policies. Our youth researchers noted that they feel pressure to “solve this problem (health inequity) that has taken generations to deteriorate.” Getting input from youth voices earlier may help in some of this deterioration of health equity.

Future research is needed that elevates the voices of those most marginalized (such as youth of color) by healthcare systems regarding their healthcare priorities and recommendations, especially in the context of COVID‐19. Participatory research methods are critical as they promote the equitable participation and leadership of community members in the development of knowledge and solutions (Teixeira et al., 2021).

### 4.2 Strengths and limitations

The project was designed to be held in person; however, due to the restrictions of the COVID‐19 pandemic, the activities were conducted exclusively on Zoom. While being online resulted in benefits in terms of all youth members attending and participating in each of the sessions (e.g., reduced travel time and weather concerns), there were slight adaptations made to adhere to the online platform. For example, the photovoice sessions were conducted in a condensed time frame so each participant independently reflected on their own photos using the SHOWed prompts in advance of the group sessions, rather than doing it as an exercise in the session, which could have resulted in a less collective group analysis of the photos. That said, the youth had multiple opportunities to collectively reflect on the photos, key themes, and group narrative. Further, the use of symbols to vote online was confusing for some youth and resulted in one youth's votes initially not being visible to others. This error was corrected, and all votes were included in the final tally.

Another limitation of holding the project online was that the youth researchers were not able to carry out robust dissemination and action plans. Ideally, they would have presented their photos in person through a photo exhibition or other forum to stakeholders in the hospital and community with the goal of influencing policy and/or practice. To adhere to the online format, the youth presented their findings as part of the plenary session at the Boston Area Research Initiative's (BARI) Build Back Better Conference which included an audience of policymakers, researchers, and community‐based agencies, and community members. They also presented their research recommendations online to stakeholders at Boston Medical Center Family Medicine Department's monthly meeting. The degree to which their recommendations resulted in policy or program impacts is unknown, but is an important area of future research.

## 5 CONCLUSION

Through photovoice methods, youth identified multilevel barriers to healthcare access and utilization in communities of color. In particular, the institutional racism underpinning health policy and programs. To address medical mistrust and promote health equity, it is critical to engage youth voice and leadership in healthcare practice and innovation. This project shows the importance of using participatory research methods, such as photovoice, in researching health equity. These participatory methods provide a rich and complex understanding of individual community perspectives in an accessible way that elevates the voices of those individuals that are most impacted but have been historically marginalized in research. Participatory methods provide youth and communities of color with opportunities to define priorities, engage in data collection and analysis, and develop youth‐driven recommendations.

## CONFLICTS OF INTEREST

The authors declare no conflicts of interest.

### PEER REVIEW

The peer review history for this article is available at https://publons.com/publon/10.1002/jcop.22866.

## Data Availability

Research data are not shared.

## Footnotes

1 The youth research team selected the term “youth of color,” in keeping with the self‐identification of the youth members participating in this project. The eight youth members identify as Latinx/Hispanic (2), Vietnamese‐Chinese (1), Black/Asian (1), Black/African American (2), Black/Nigerian‐American (1), and Afro‐Caribbean (1). Given that there are no indigenous youth members in this YPAR project, the research team in consultation with the youth determined the term “youth of color” to be more accurately representative of the YPAR youth members than the term Black, Indigenous, and Youth of Color. The research team acknowledges the limitations of the term “youth of color” in that it may contribute to a flattening or collapsing of diverging experiences and contexts that youth from different racial and ethnic backgrounds may experience.
